# Laparoscopic Nephroureterectomy with Heminephrectomy for Urothelial Carcinoma of the Upper Renal Pelvis on the Left Side of the Horseshoe Kidney

**DOI:** 10.1155/2022/4985041

**Published:** 2022-06-21

**Authors:** Taro Ikeda, Kazunori Matsumoto, Go Hasegawa, Yohei Ikeda, Noboru Hara, Tsutomu Nishiyama

**Affiliations:** ^1^Department of Urology, Uonuma Institute of Community Medicine, Niigata University Medical and Dental Hospital, Minamiuonuma, Niigata, Japan; ^2^Department of Radiation Engineering, Uonuma Institute of Community Medicine, Niigata University Medical and Dental Hospital, Minamiuonuma, Niigata, Japan; ^3^Department of Pathology, Uonuma Institute of Community Medicine, Niigata University Medical and Dental Hospital, Minamiuonuma, Niigata, Japan; ^4^Department of Diagnostic Radiology, Uonuma Institute of Community Medicine, Niigata University Medical and Dental Hospital, Minamiuonuma, Niigata, Japan

## Abstract

A 70-year-old male was diagnosed with urothelial carcinoma of the upper renal pelvis on the left side of the horseshoe kidney. Preoperative thin-slice contrast-enhanced CT with three-dimensional reconstruction of the images revealed that two arteries arising from the aorta supplied the left moiety of the horseshoe kidney. He underwent laparoscopic transperitoneal nephroureterectomy with heminephrectomy on the left side of the horseshoe kidney visualized by indocyanine green fluorescence system. The histopathological findings of the renal pelvic tumor revealed invasive urothelial carcinoma with squamous differentiation, high grade, and pT3.

## 1. Introduction

Upper tract urothelial carcinoma represents a small proportion (5%-10%) of all urothelial cancers [1]. Horseshoe kidney is a congenital abnormality in which the two kidneys are fused at their lower poles by a region of parenchymal and fibrous tissue called the isthmus [2]. There is no known association of horseshoe kidney with urothelial cancer; however, there are several case reports describing surgical management of tumors in patients with horseshoe kidney [3–7]. Herein, we present a case of urothelial carcinoma in the left renal pelvis of the horseshoe kidney. The patient underwent laparoscopic left nephroureterectomy with heminephrectomy for urothelial carcinoma of the upper renal pelvis on the left side of the horseshoe kidney.

## 2. Case Report

The patient, a 70-year-old male, was treated with laparoscopic radical prostatectomy and salvage radiotherapy for localized prostate cancer in 2016 and 2017.

**Figure 1:**
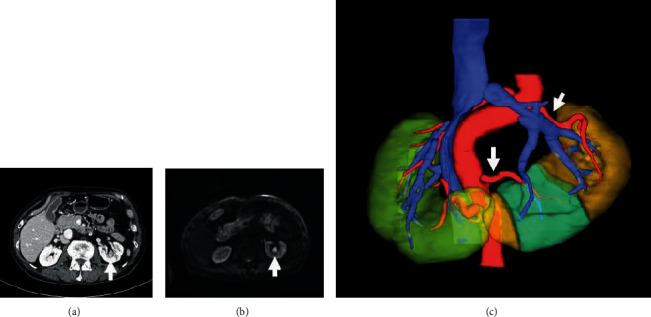
(a) CT findings. CT reveals a mass lesion of upper pelvis of the left side of the horseshoe kidney (arrow). (b) MRI findings. MRI reveals a high signal intensity on diffusion-weighted imaging (arrow). (c) Reconstruction of three-dimensional analysis of the images of thin-slice contrast-enhanced CT. Three-dimensional reconstruction reveals two renal arteries arising from the aorta that supplied to the left side of the horseshoe kidney (arrow).

Atypical urothelial cells were found in urine cytology in 2019 on a prostate cancer follow-up. There was no evidence of urothelial cancer on repeated computed tomography (CT) and cystoscopy; however, atypical urothelial cells in urine cytology continued to be detected. In May 2021, CT and magnetic resonance image (MRI) revealed a mass lesion of the upper pelvis of the left side of the horseshoe kidney (Figures 1(a) and 1(b)). A urine sample collected from the left side of the renal pelvis was suspicious for high-grade urothelial carcinoma in cytology. Thus, he was diagnosed with urothelial carcinoma of the upper renal pelvis on the left side of the horseshoe kidney. Preoperative thin-slice contrast-enhanced CT was performed with reconstruction of three-dimensional analysis of the images (SYNAPSE VINCENT, FUJIFILM, Tokyo, Japan), which revealed that two arteries arising from the aorta supplied the left moiety of the horseshoe kidney (Figure 1(c)).

### 2.1. Operational Findings

**Figure 2:**
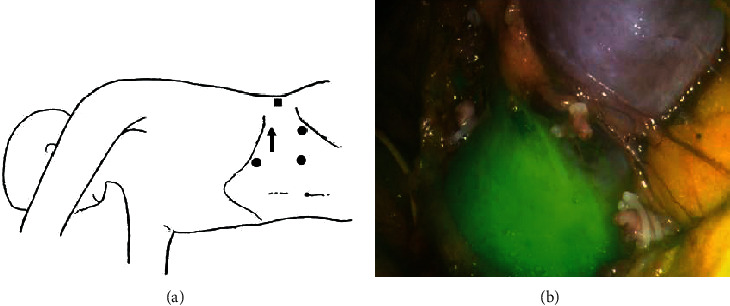
(a) The placement of the trocar port and an incision for removal of the extirpating organs. Black circle: operator's port. Black triangle: camera port. Black square: assistant's port. Black rectangle: six cm incision for removal of extirpating organs. (b) Interruption of blood flow is confirmed between the left moiety and the right moiety visualized by indocyanine green (ICG) fluorescence system. The green region shows blood flow to the right moiety.

The patient underwent laparoscopic transperitoneal nephroureterectomy with heminephrectomy on the left side of the horseshoe kidney in the right decubitus position in July 2021 (Figure 2(a)). We ligated two arteries with arising from the aorta that supplied the left moiety using Hem-o-loc (Teleflex Incorporated, Morrisville, North Carolina, USA). We confirmed interruption of blood flow to the left moiety and blood flow to the right moiety visualized by indocyanine green (ICG) fluorescence system (PINPOINT Endoscopic fluorescence imaging system, Stryker, Michigan, USA) (Figure 2(b)). Then, we ligated the vein of the left moiety flowing into the vena cava with Hem-o-loc. A splitting operation was performed on the renal tissue without blood flow between the interruption of blood flow to the left moiety and blood flow to the right moiety. Little bleeding was encountered during and after the separation. The ureter was ligated with Hem-o-loc not to implant cancer cells in lower urinary tract following confirming left ureter. After performing left heminephrectomy, we performed an en-bloc left nephroureterectomy including the left ureter close to the bladder cuff. We cut the ureter at the tenting site of ureter at the vesicoureteral junction laparoscopically without opening the bladder. The excised organ was removed from the abdominal space. The operating time was six hours 21 minutes and estimated total blood loss was 250 ml. No postoperative complication occurred.

### 2.2. Pathological Findings (Figure 3)

The excised organ revealed the tumor was a 30 × 15 mm mass in the upper renal pelvis. The histopathological findings of the renal pelvic tumor revealed invasive urothelial carcinoma with squamous differentiation, high grade, and pT3.

He was discharged from the hospital on postoperative four days. In February 2022, there was no evidence of recurrence.

## 3. Discussion

Horseshoe kidney represents one of the most frequent renal malformations, with an incidence of 0.25% among the general population, being twice as frequent in males [2]. The fusion prevents normal rotation of the kidney so that the ureter arises anteriorly and crosses over the isthmus.

It has been reported that the incidence of carcinoma of the renal pelvis is at least three times greater in horseshoe kidney patients [3]. The higher incidence has been attributed to chronic noxious stimulation associated with infection, stasis, and stones; however, the pathological features are unclear. Mizusawa et al. reviewed 24 cases of renal pelvic tumor in horseshoe kidneys previously reported in Japan [8]. Seven cases (30%) included components of squamous cell carcinoma. The renal pelvic tumor of the present patient also showed invasive high-grade urothelial carcinoma with squamous differentiation. Voided urine cytology performances are worse for upper urinary tract urothelial carcinoma than for urothelial carcinoma of bladder [1]. Because of its low sensitivity, urine samples are collected from the upper urinary tracts before injection of contrast medium for retrograde pyelography.

Variable blood supply is one of the anatomical features of horseshoe kidney. Majos et al. reported that horseshoe kidneys had nearly twice as many renal arteries as normal kidneys (4.57 vs. 2.4 per patient) [9]. Preoperative thin-slice contrast-enhanced CT with reconstruction of three-dimensional analysis of the images is extremely useful for detailed analysis of blood supply. We used a three-dimensional analyzer of the images. Intraoperative ICG fluorescence navigation system is used for laparoscopic partial nephrectomy to conserve more normal renal tissue without increasing the rate of positive surgical margins [10]. Moreover, we revealed that blood flow evaluation by ICG system is also useful for performing laparoscopic heminephrectomy for a horseshoe kidney. We have not been detected previously published case reports of upper urinary tract cancer arising from horseshoe kidney treated with laparoscopic nephroureterectomy with 3D-reconstructed images or intraoperative ICG fluorescence navigation system.

## 4. Conclusion

We performed laparoscopic transperitoneal nephroureterectomy with heminephrectomy for urothelial carcinoma of the renal pelvis on the left side of the horseshoe kidney. Preoperative thin-slice contrast-enhanced CT with reconstruction of three-dimensional analysis of the images and intraoperative ICG fluorescence navigation system were useful for performing heminephrectomy.

## Figures and Tables

**Figure 3:**
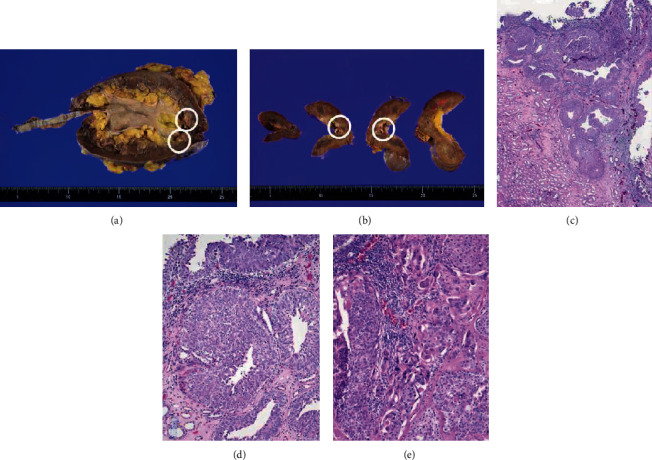
Pathological findings. The macroscopic specimen reveals the tumor was a 30 × 15 mm mass in the upper renal pelvis ((a, b), circle). The histopathological findings of the renal pelvic tumor reveal invasive high grade urothelial carcinoma (c, d) with squamous differentiation (e). The tumor invades renal papilla and there is no lymphovascular invasion.
